# Hospital-acquired infections worldwide in relation to Gross Domestic Product: A global ecological analysis of point-prevalence surveys

**DOI:** 10.1371/journal.pgph.0006998

**Published:** 2026-08-13

**Authors:** Emma Etcheverry, Philippe Vanhems, Juliette Dessemon, Christelle Elias, Mitra Saadatian-Elahi

**Affiliations:** 1 CIRI, Centre International de Recherche en Infectiologie, Équipe Santé Publique, Épidémiologie et Écologie Évolutive des Maladies Infectieuses (PHE3ID), Inserm U1111, CNRS UMR5308, ENS de Lyon, Université Claude Bernard Lyon 1, Lyon, France; 2 Service d’Hygiène, Epidémiologie, Infectiovigilance et Prévention, Hopital Edouard Herriot, Hospices Civils de Lyon, Lyon, France; The University of Sydney, AUSTRALIA

## Abstract

Hospital-acquired infections (HAI) are a major public health problem worldwide. The World Health Organization estimates that 7% of patients could be affected by at least one HAI in high income countries (HICs), and 15% in low- and middle-income countries (LMICs).We hypothesized that countries with higher Groos Domestic Product (GDP) would have lower HAI prevalence rates, reflecting better investment in infection prevention and control programs.We therefore carried out an ecological study, based on published results from national or multicenter repeated prevalence surveys of HAI, to describe the relationship between GDP by country and the HAI prevalence rate. Economic resources per country were estimated using the GDP. Univariate correlation was calculated and presented graphically. The study included 94 HAI prevalence surveys conducted in 19 countries between 1984 and 2022. The majority of surveys (76.6%) were carried out in HICs. The number of surveys increased from 9 between 1984 and 1993–38 between 2014 and 2022. The weighted average proportion of patients with at least one HAI was 6.1%. We observed a decreasing trend in the weighted average proportion of patients with at least one HAI as GDP per capita increased (R = - 0.3997; P = 0.09).This ecological study provides insights into a potential association between national economic level and the burden of HAIs. To better investigate the impact of GDP on HAI prevalence, future research should incorporate a larger number of surveys with more balanced representation across geographic regions and income levels.

## Introduction

Healthcare-associated infections (HAI), defined as infections that occur “*while receiving health care, developed in a hospital or other health care facility that first appear 48 hours or more after hospital admission, or within 30 days after having received health care*”, represent a major global health problem and a challenge for health practitioners worldwide [1]. The most common HAI worldwide are catheter-associated urinary tract infections, followed by surgical site infections [2]. The main known risk factors are age, immunosuppression, admission to an intensive care unit (ICU), length of hospital stay, surgical procedures and use of medical devices [3]. In low and medium-income countries (LMICs), additional factors such as poor hygiene, and sanitation due to limited economical resources could further increase the risk of HAI [3]

HAI contribute to significant morbidity, mortality and financial burden on health care systems [3]. With an annual estimation of 136 million antibiotic resistant HAIs, these infections are also a major driver of antimicrobial resistance, predominantly in lower income countries [4].

The World Health Organization (WHO) estimates that the prevalence of hospitalized patients with at least one HAI was around 7% in high-income countries (HICs), and around 15% in LMICs [4]. The burden raise even more in ICUs, where up to 30% of patients may develop HAI, with incidence rates reported to be much higher in LMICs [4]. WHO estimates suggest a mortality rate of around 10% among affected patients**,** although precise LMIC-specific mortality estimates remain difficult to quantify. Studies from HICs report crude mortality rates of 12–80%, with 37,000 deaths directly attributable in Europe [3]. The poor understanding of the real burden of HAIs in LMICs is mainly due to the lack of effective surveillance and reporting [3]. Limited laboratory capacity, inadequate infection and control prevention infrastructure, and insufficient trained personnel are among factors that could explain underdeveloped national surveillance networks in LMICs that contribute to under-detection and under-reporting of HAIs [4].

Improving economic conditions and healthcare financing, particularly through better investment in water, sanitation, and hygiene in healthcare facilities, can reduce healthcare-associated infections [5]. Gross domestic product (GDP) per capita is often used as an indicator of a country’s economic strength [6] and can serve as an important indicator for evaluating the performance and capacity of a healthcare system. Indeed, GDP per capita can influence the resources available for healthcare delivery, infection control, surveillance, and public health infrastructure [7]. A correlation between higher GDP per capita and increased healthcare spending often reflects greater investment in medical infrastructure and advanced technologies. Conversely, disparities between GDP growth and healthcare expenditure may indicate inefficiencies, or unequal access within the healthcare system. Therefore, analyzing this correlation provides valuable insights into the prioritization, and overall effectiveness of healthcare systems [8].

On the other hand, HAIs are widely regarded as an indicator of patient safety and healthcare quality because presence of strong infection prevention and control practices, surveillance systems, and trained staff can reduce considerably their occurrence.

While economic disparities are recognized as important determinants of HAI burden, few studies have examined the relationship between GDP per capita and HAI prevalence at the country level. We hypothesized that countries with higher GDP would have lower HAI prevalence rates, reflecting better investment in infection prevention and control programs. Building on this framework, we carried out an ecological study using published HAI prevalence surveys to examine the association between GDP and the prevalence of HAIs and to explore how macroeconomic conditions may influence infection burden within healthcare systems.

## Materials and methods

We conducted an exploratory ecological study using secondary quantitative data obtained from published national HAI point prevalence surveys (PPS). HAI was defined as the outcome variable and GDP as the explanatory variable. To be included in the analysis, countries had to have performed at least two published multicenter HAI-PPS at national level between 1984 and 2022 (S1 File). Countries were classified according to the WHO classification (Europe, America, Western Pacific, Southeast Asia, Eastern Mediterranean) [9] (Table 1).

**Table 1:** Published point prevalence according to the WHO regions and World Bank Group (WBG) classification.

Country	Number of survey	WBG Classification	Reference
America			
Argentina	11	Upper middle	[10,11]
Canada	3	High	[12]
Cuba	2	Lower middle	[13,14]
United States	2	High	[15,16]
Europe			
Germany	3	High	[17–19]
Belgium	4	High	[20–22]
Spain	18	High	[23–25]
France	6	High	[26–31]
Greece	2	High	[32,33]
Italy	5	High	[34–38]
Norway	3	High	[39–41]
Netherlands	10	High	[42]
Slovenia	3	High	[43–45]
Switzerland	9	High	[46–49]
Eastern Mediteranean			
Tunisia	2	Lower middle	[50,51]
Southeast Asia			
Thailand	4	Lower middle	[52–55]
Western Pacific			
Australia	2	High	[56,57]
China	3	Upper middle	[58–60]
Japan	2	High	[61,62]

The WBG income classification was used to categorize countries according to their income level, including high-income, upper-middle-income, and lower-middle-income groups. This classification, based on GDP per capita, provides a standardized framework for comparing countries with different economic capacities and allows for more meaningful interpretation of variations in healthcare outcomes and expenditures across income strata [63]. Country-level data on GDP per capita, expressed as $US, were obtained from the WBG database [64]. For each country, the average GDP per capita was calculated as the average of GDPs for the years in which the surveys were conducted.

The number of participating facilities, the total number of patients included in each PPS and the weighted average proportion of patients with at least one HAI were retrieved from the published studies. Based on the WHO region and the WBG classification, the average prevalence weighted by the number of patients included per survey was then calculated for each country.

### Statistical analysis

A descriptive analysis was conducted using the median and interquartile range (IQR). The Pearson correlation coefficient was calculated to assess the association between HAI prevalence and GDP per capita. To examine temporal trends, observation periods were stratified into 10-year intervals (1984–1993, 1994–2003, 2004–2013, and 2014–2022), allowing for the analysis of changes in HAI prevalence over time and the estimation of average trends across sufficiently long periods.

For each WHO region and for each country, the average HAI prevalence, weighted by the number of patients included in each survey, and the corresponding average GDP per capita were calculated as follow:


$$\begin{array}{c} Weighted\;average\;prevalence\;=\;\frac{\sum \;(\text{prevalence reported in survey}\times \text{number of patients included in survey})}{\sum \text{number of patients included in survey}} \end{array}$$

$$\begin{array}{c} Average\;GDP\;per\;capita=\;\frac{\sum \text{ GDP per capita for the year of survey}}{\sum \text{ number of surveys conducted in the region or in the country}} \end{array}$$

All analyses were performed using R-Studio software, version 4.2.

## Results

Overall, 94 prevalence surveys were conducted in 19 countries between 1984 and 2022. Characteristics of HAI prevalence surveys by WHO region and by WBG classification are presented in Table 2. The large majority of surveys (n = 63; 67.0%) were conducted in Europe, 19.1% in the Americas, 7.4% in the Western Pacific, 4.3% in Southeast Asia and 2.1% in the Eastern Mediterranean region. Based on the WBG classification, 76.6% of the countries were considered as HICs, 14.9% were upper-middle-income and 8.5% LMICs.

**Table 2:** Characteristics of HAI prevalence surveys by WHO region and by WBG classification.

	GDP per capita ($)* (N = 94)	Number of participating facilities (%)* (N = 94)	Number of patients included* (N = 94)	Number of people infected with at least one HAI* (N = 94)	Weighted average proportion of patients with at least one HAI (%)
WHO region					
America (N = 18)	12,964 (11,107)	100 (111)	5,288 (2,356)	435 (210)	8.2
Europe (N = 63)	36,470 (22,964)	76 (222)	19,026 (44,192)	994 (3,335)	6.4
Eastern Mediterranean (N = 2)	3,670 (845)	105 (39)	7,857 (792)	519 (55)	6.6
Southeast Asia (N = 4)	1,924 (598)	28 (13)	10,119 (3,294)	777 (164)	7.4
Western Pacific (N = 7)	39,751 (8,374)	20 (149)	10,209 (38,308)	387 (1,629)	3.8
WBG classification					
High (N = 73)	38,715 (23,275)	71 (214)	16,672 (43,032)	944 (3,051)	6.4
Upper middle (N = 14)	12,964 (2,477)	121 (90)	4,834 (1,789)	445 (212)	4.2
Lower middle (N = 8)	2,706 (1,418)	33 (21)	7,857 (3,350)	608 (314)	7.1

* Median (IQR).

The overall median (IQR; min–max) annual GDP per capita was $29,628 (20,832; 1,155–93,446), with Southeast Asia having the lowest annual GDP per capita. The overall median (IQR; min-max) number of participating healthcare facilities by survey was 68.5 (366.8; 2–2,337) with Japan and France having the min and max numbers of involved facilities respectively. The overall median number of patients included in the PPS was 13,344 (72,886; 820–407,208). The overall weighted average proportion of patients with at least one HAI included per survey was 6.1% (N = 94). The highest rates were observed in the WHO region of the Americas (8.2%), followed by Southeast Asia (7.4%), the Eastern Mediterranean (6.6%) region, Europe (6.4%), and the Western Pacific (3.8%) (Table 2).

**Fig 1:**
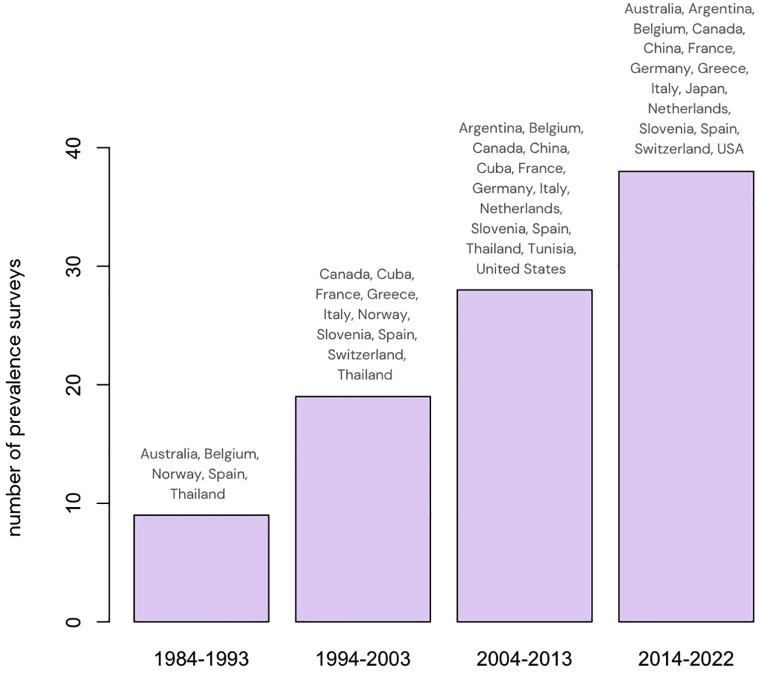
Number of published prevalence surveys over time (N = 94).

The number of published surveys increased overtime rising from 9 surveys between 1984 and 1993–38 surveys between 2014 and 2022 (Fig 1).

**Fig 2:**
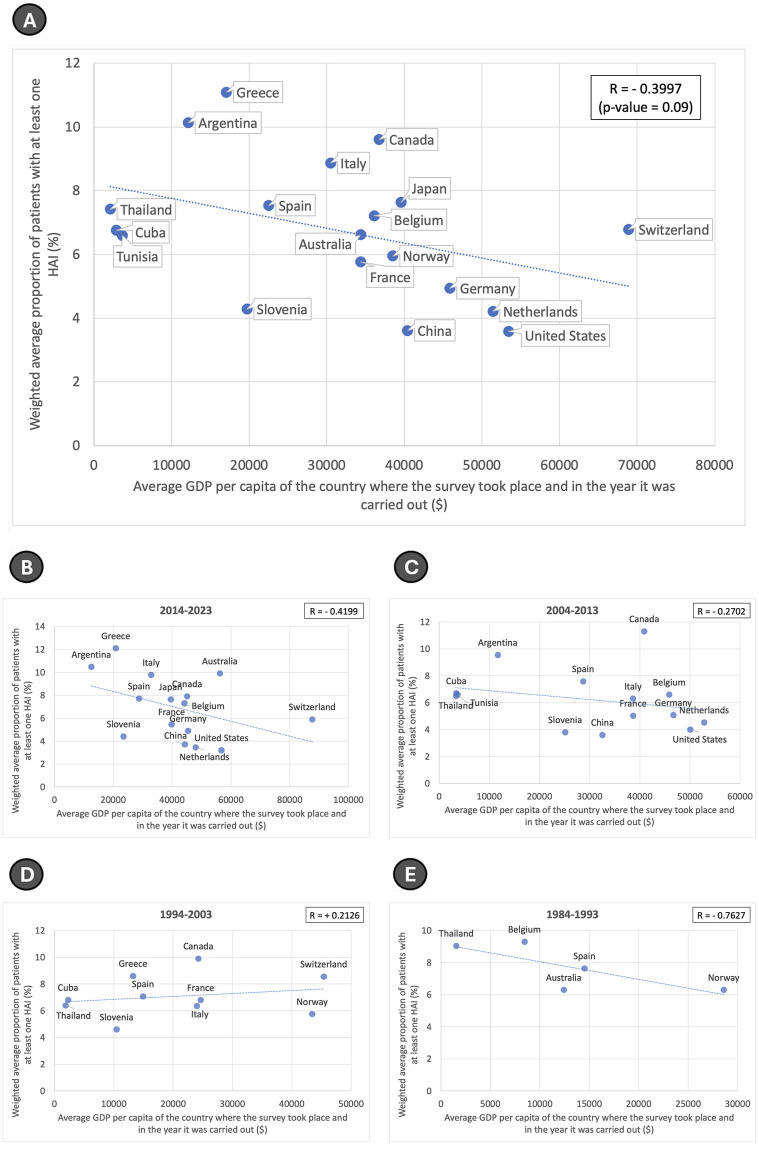
Weighted average proportion of patients with at least one HAI per country as a function of the average GDP per capita of the country.

The weighted average proportion of patients with at least one HAI per country plotted against average GDP per capita from 1984 to 2023 and stratified by 10-years interval is represented in Fig 2. The lowest weighted average proportion of patients with at least one HAI was found in the USA (3.6%) with an average GDP of $53,414, and the highest in Greece (11.1%) with an average GDP of $17,058. The weighted average proportion of patients with at least one HAI tended to decrease with increasing average GDP per capita (R = -0.3997; P = 0.09). The strongest association between GDP and prevalence rates was observed in 1984–1993 (R = 0.7627) and the lowest in 2014–2023 (R = - 4199). The trend over time in the average prevalence rate according to GDP showed stronger association in LMICs (R = -0.6224) as compared to HICs (R = - 04325; S1 Fig).

## Discussion

The primary objective of this ecological study was to assess the potential relationship between a country’s economic level, estimated by average GDP per capita, and the prevalence of HAIs as reported in published PPS.

Overall, we observed a decreasing but not statistically significant trend in the weighted average proportion of patients with at least one HAI as GDP per capita increased. Our findings are consistent with a recent meta-analysis on the global burden of HAIs, which reported substantially higher HAI prevalence in LMICs (32%) compared with HICs (6%) [65]. Although the specific outcome measures differed between our study and the mata-analysis, GDP can be considered an indirect measure of a country’s income classification. We can therefore suggest that higher levels of economic development may be associated with less HAI occurence. The higher prevalence of HAIs in LMICs is influenced by multiple factors, including inadequate healthcare infrastructure, limited sustainability of surveillance systems, limited number of trained infection prevention and control personnel, and broader poverty-related constraints [66].

Stratification of HAI prevalence rates by 10-year intervals and by income category showed that the strongest associations between GDP and HAI prevalence were observed during 1984–1993 and 2004–2013. Stratification by income category further suggested a stronger association between GDP and HAI prevalence in LMICs. The variation in the strength of the association between GDP and HAI prevalence over time may reflect several factors, including differences in the number of surveys conducted and changes in the quality and completeness of surveillance data. Advances in medical technology and healthcare delivery may also have improved infection prevention and control practices, thereby modifying the relationship between GDP and HAI prevalence over time. In the earlier decades, particularly the 1980s, GDP may have been a stronger proxy for healthcare infrastructure, quality of care, and infection control capacity. This stronger association in LMICs may therfore reflect a saturation effect in the relationship between GDP and HAI prevalence. At lower levels of economic development, increases in GDP are likely to be accompanied by substantial improvements in healthcare infrastructure and infection prevention capacity. In contrast, once countries achieve higher levels of healthcare investment and system maturity, further economic growth may provide only marginal benefits for reducing HAI burden. This could explain the progressively weaker association observed across income groups and suggests that GDP may be a more informative marker of HAI risk in LMICs than in higher-income settings. However, these findings should be interpreted cautiously, as the relatively small number of LMIC studies may have limited the stability and generalizability of the estimates.

According to a WHO report, the implementation of infection prevention and control programs has been associated with significant reductions in HAI rates, ranging from 35% to 70% [67]. These programs are based on serval fundamental components, including guidelines, training and education, surveillance, multimodal strategies, monitoring and evaluation, adequate staffing, and appropriate infrastructure. Indeed, existance of such programs enable implementation of practical interventions such as promoting hand hygiene, cleaning the environment, and providing personal protective equipment [67]. We also observed an increasing number of published HAI prevalence surveys over time. This trend may be partly attributable to the expansion of infection prevention and control programs, particularly following the COVID-19 pandemic. Data from the WHO global surveys conducted in 62 countries during 2017–2018 and 2021–2022 demonstrated a marked increase in the proportion of countries with a dedicated budget for such programs, from 25.8% to 73.6%. Similarly, the percentage of countries with ongoing infection prevention and control programs rose from 58.1% to 85% [68]. These developments likely contributed to enhanced surveillance capacity and increased reporting of HAI prevalence.

This study has several strengths. The use of data from national prevalence surveys enhances reliability, as these surveys are based on objective measurements derived from medical records and generally follow standardized methodologies. Another major strength is the extended observation period. Aggregating multiple cross-sectional surveys over several decades allows for the identification of long-term trends and facilitates analysis of the evolution of HAI prevalence over time.

However, some limitations should be considered when interpreting these findings. First, the unequal representation of countries by income level, particularly the scarcity of published data from LMICs, may introduce information bias. Income level may also influence both the likelihood of conducting prevalence surveys and the reported burden of HAIs, thereby affecting study availability and reducing statistical power. The underepresantaion of LMICs limit the interpretability of the findings and the stratification ability and to explain the saturation phenomenon. The HAIs rates being extracted from published littérature, we used secondary quantitative data to detremine the correlation between HAI rate and GDP. As a consequence, HAI prevalence could be underrepresented in the primary studies from developing countries and the reported findings could be subject to measurement limitations and reporting biases. Although the correlation did not reach the conventional threshold for statistical significance (p < 0.05), the observed trends remain relevant in the context of exploratory analysis. As an ecological study, our analysis is based on correlations rather than causal inference. In addition, methodological heterogeneity arising from the use of different PPS protocols worldwide (e.g., G-PPS, WHO PPS, ECDC PPS, and MURIA) limits comparability across settings.

In conclusion, this ecological study provides insights into a potential association between national economic level and the burden of HAIs. To better investigate the impact of GDP on HAI prevalence, future research should incorporate a larger number of surveys with more balanced representation across geographic regions and income levels. Strengthening systematic and regular global surveillance of HAIs through well-designed initiatives such as the Global Point Prevalence Survey of Antimicrobial Use and Resistance [69] and the EPIC studies [70–72] would further support this objective and contribute to improved understanding and control of HAIs worldwide.

## Supporting information

S1 Fig Weighted average proportion of patients with at least one HAI per country stratified by income level. HAI: Health care associated infection; GDP: Gross domestic product; R: Pearson’s correlation coefficient.
(TIFF)

S1 File Data. (XLSX)
